# Intratumoral *Bacillus* regulating colorectal cancer liver metastasis: links to lipid metabolism and APOE^+^ macrophages

**DOI:** 10.3389/fimmu.2026.1938606

**Published:** 2026-08-28

**Authors:** Juhang Chu, Haitao Huang, Yuheng Hu, Qiaoer Wang, Tao Yuan, Shengyu Huang, Jianbo Lin, Chenxiang He, Haoyu Li, Hao Shen, Jun Li

**Affiliations:** 1 Department of Hepatobiliary and Pancreatic Surgery, the Tenth People’s Hospital of Tongji University, School of Medicine, Tongji University, Shanghai, China; 2 Department of Emergency, Changhai Hospital, Naval Medical University, Shanghai, China; 3 Clinical Research Institute and Department of Hepatobiliary Surgery, Eastern Hepatobiliary Surgery Hospital and National Centre for Liver Cancer, Naval Medical University, Shanghai, China

**Keywords:** APOE^+^ macrophages, Bacillus, colorectal cancer liver metastasis, intratumoral microbiota, lipid metabolic regulation, metabolomics, single-cell transcriptomics, tumor immune microenvironment

## Abstract

**Introduction:**

Colorectal cancer liver metastasis (CRLM) develops in a hepatic niche where tumor cells encounter organ‑specific microbial, metabolic, and immune constraints. The role of intratumoral bacteria in this metastatic ecosystem remains incompletely defined.

**Methods:**

We analyzed tumor and peritumoral tissues from 20 patients with non‑metastatic colorectal cancer (CRC) and 20 patients with CRLM by 5R 16S rDNA sequencing, untargeted metabolomics, and functional assays. Public datasets were used for single‑cell analysis of the tumor immune microenvironment.

**Results:**

Primary colorectal lesions and paired liver metastases showed distinct microbial structures. Liver metastatic lesions had lower microbial diversity, and 73 genera differed between CRLM primary tumors and liver metastases. *Bacillus* was enriched in metastatic tumors and was associated with fatty‑acid‑related microbial pathways, serum CEA and CA19‑9 levels, and lipid‑related metabolites that accumulated in liver metastases. Bacillus supernatant increased SW480 and SW620 cell migration and proliferation *in vitro*, with the strongest activity at a multiplicity of infection (MOI)‑equivalent of 20. In an intrasplenic CRLM model, viable *Bacillus* and Bacillus supernatant increased metastatic tumor growth, whereas antibiotic intervention reduced this effect. Metabolomic profiling detected enrichment of lipids and lipid‑like molecules, including carnitine‑ and bile‑acid‑related metabolites, in liver metastatic lesions. Single‑cell analysis of public datasets showed remodeling of the tumor immune microenvironment (TIME) and identified APOE⁺ macrophages as lipid‑associated myeloid cells with broad ligand‑receptor communication with epithelial, immune, and stromal compartments.

**Discussion:**

Together, these findings provide evidence that *Bacillus* promotes metastatic phenotypes and reveal associations among intratumoral *Bacillus*, lipid‑related metabolic alterations, and APOE^+^ macrophage‑associated communication signatures in CRLM.

## Introduction

The immune and stromal ecosystems surrounding colorectal tumors shape tumor growth, dissemination, and treatment response (1). Liver metastasis represents a common and clinically demanding pattern of colorectal cancer (CRC) progression. Recent studies describe colorectal cancer liver metastasis (CRLM) as a process driven by tumor-cell plasticity, hepatic niche adaptation, and reciprocal crosstalk between disseminated epithelial cells and the liver microenvironment (2–4). Surgical resection, systemic chemotherapy, targeted agents, and immunotherapy have improved care for selected patients, but long-term disease control remains limited for many patients with CRLM. A better understanding of the microbial, metabolic, and cellular interactions that support hepatic colonization is needed to identify actionable vulnerabilities in this setting.

Intratumoral microbiota is now viewed as an active part of the tumor ecosystem rather than a passive contaminant. Tumor-resident microbes can alter malignant progression by reshaping metabolism, modifying immune responses, influencing spatial heterogeneity, and affecting therapeutic response (5–7). In CRC, tumor microbial profiles have been associated with clinicomolecular subtypes, disease progression, and patient prognosis (8–11). Most available evidence, however, centers on primary colorectal tumors. The microbial structure of liver metastatic lesions, and the contribution of metastasis-enriched bacteria to local metabolic and immune adaptation, remain less clear.

CRLM offers a useful model for studying how tumor-associated microbes adapt to a new organ environment. Colorectal tumor cells that enter the liver are exposed to bile acids, hepatic resident macrophages, immune surveillance, and metabolic constraints that differ from the intestinal niche. These features raise two related questions. Primary tumors and paired liver metastases may differ in intratumoral bacterial composition. Metastasis-enriched bacteria may also influence the lipid metabolic and immune-cell programs that support metastatic growth. A multi-layered analysis across microbial, metabolic, and host-cell compartments is needed to address these questions.

We integrated 5R 16S rDNA sequencing, untargeted metabolomics, public single-cell RNA sequencing (scRNA-seq) analysis, and functional experiments to compare colorectal primary tumors with liver metastatic lesions. The study first examined intratumoral microbial diversity and taxonomic composition across primary and metastatic sites. Metastasis-enriched bacterial genera were then evaluated in relation to predicted microbial functions, tissue metabolites, and clinical indicators. Functional assays tested whether *Bacillus*-derived products altered CRC cell migration, proliferation, and metastatic tumor growth. Single-cell analysis was used to define host immune remodeling in CRLM, with a focus on lipid-associated apolipoprotein (APOE)^+^ macrophages and their ligand-receptor communication programs. This design allowed us to explore potential associations among microbial, metabolic, and immune features within the CRLM niche.

## Materials and methods

### Patient selection, sample collection, and retrospective data collection

Patients diagnosed with CRC at the Tenth People’s Hospital of Tongji University between January 2023 and December 2024 were retrospectively screened. The initial screening set included 105 patients. Eligible patients had newly diagnosed CRC or CRLM, histological confirmation of CRC by colonoscopic biopsy, and, for the CRLM cohort, liver metastatic lesions detected by enhanced CT or PET-CT. Patients were excluded if they had undergone colorectal resection before sampling, received chemotherapy, targeted therapy, or immunotherapy, received antibiotics (Abx) within 3 months before surgery, had metastases in organs other than the liver, or had another malignancy involving the colon or rectum. After screening, 20 patients with non-metastatic CRC and 20 patients with CRLM were included.

All included patients underwent surgical treatment. Tumor tissues and adjacent nontumor tissues were collected under aseptic conditions for multi-omics analyses. Clinical information, laboratory indices, and pathological findings were retrieved from medical records. All procedures followed the regulations of the National Health Commission of China and the principles of the Declaration of Helsinki. Each participant provided written informed consent. The study protocol was approved by the Institutional Review Board of Tenth People’s Hospital Affiliated with Tongji University and was registered at ClinicalTrials.gov (NCT07509398)) for patient enrollment.

### 5R 16S rDNA sequencing and microbial analysis

A total of 120 tissue specimens from six anatomical groups were analyzed: CRC lesions, adjacent nontumor tissues from non-metastatic CRC, CRLM primary tumors, adjacent tissues from CRLM primary tumors, paired liver metastatic lesions, and adjacent liver tissues. Five blank tubes were included as sequencing quality-control controls. In addition, DNA extraction negative controls and PCR no-template controls were included to monitor potential contaminants introduced during DNA extraction and PCR amplification, respectively. Taxa exhibiting a prevalence higher than 30% in negative controls were defined as experimental artifacts and rigorously filtered from all biological samples using standard tumor microbiome decontamination pipelines (12). The same contamination-filtering criterion was applied consistently to samples from both primary colorectal tumors and liver metastatic lesions before the comparison of microbial profiles between the two anatomical sites. The V2, V3, V5, V6, and V8 regions of the bacterial 16S rDNA were amplified and sequenced on an Illumina NovaSeq 6000 platform by Hangzhou Lianchuan Biotechnology Co., Ltd.

Filtered reads were mapped to amplified regions, integrated with the short multiple-region framework (SMURF), and annotated against the GreenGenes database after contaminant signals were removed (13). Alpha diversity was calculated in quantitative insights into microbial ecology (QIIME) and displayed with R-based boxplots. Beta diversity was assessed from Bray-Curtis and Jaccard distances calculated with the vegdist package, and permutational multivariate analysis of variance was used to test group separation. linear discriminant analysis effect size (LEfSe) analysis identified differentially abundant taxa using a linear discriminant analysis (LDA) score > 3 and P < 0.05. Phylogenetic investigation of communities by reconstruction of unobserved states 2 (PICRUSt2) inferred microbial functional pathways from GreenGenes-derived sequences, and pathway differences were evaluated by t-tests.

### ScRNA-seq data processing

Public scRNA-seq data from primary CRC lesions and matched liver metastases were obtained from GSE178318. Raw count matrices and metadata were processed with Seurat in R. Low-quality cells were removed according to the number of detected genes, total UMI count, and mitochondrial transcript percentage. DoubletFinder was used to detect and remove doublets. Genes detected in fewer than three cells were excluded before downstream analysis.

The retained expression matrices were normalized and variance-stabilized with the standard Seurat workflow. Highly variable genes were selected for principal component analysis. Patient- and sample-level batch effects were corrected during integration. Integration anchors were identified using the Seurat FindIntegrationAnchors function based on shared highly variable genes, followed by IntegrateData to correct patient- and sample-associated technical variation. Tissue origin and sample identity were retained as metadata variables to ensure preservation of biological differences between primary and metastatic lesions. The integrated object was subjected to graph-based clustering, and uniform manifold approximation and projection (UMAP) was used to visualize the cellular transcriptomic landscape.

### Cell-type annotation and tumor immune microenvironment analysis

Major cell compartments were annotated using canonical lineage markers and published CRC single-cell references. T cells were defined by CD3D, CD3E, and TRAC; B cells by MS4A1 and CD79A; plasma cells by JCHAIN and immunoglobulin genes; myeloid cells by LYZ, S100A8, and S100A9; NK cells by NKG7 and GNLY; epithelial cells by EPCAM and KRT-family genes; endothelial cells by PECAM1 and VWF; fibroblasts by COL1A1, COL1A2, and DCN; and mast cells by TPSAB1 and TPSB2. Cell-type proportions were calculated for each sample and compared between primary colorectal tumors and liver metastatic lesions. Wilcoxon testing was used for group comparisons, with paired testing applied when matched primary-metastatic samples were available.

Myeloid cells were extracted for a separate analysis. The myeloid subset was re-normalized, variable genes were recalculated, and dimensionality reduction and clustering were repeated. Subpopulations were annotated using CLEC9A for cDC1, CD1C for cDC2, FCN1 for monocytes, LAMP3 for mature regulatory dendritic cell (DCs), LILRA4 for pDCs, and APOE, ABCG1, CD163, and CD68 for APOE^+^ macrophages. The APOE^+^ macrophage population was designated Mac_APOE.

Genes enriched in Mac_APOE cells relative to other myeloid cells were identified with the Wilcoxon rank-sum test. Genes with positive log-fold changes and statistical significance were used for functional analysis. Gene Ontology biological process terms and hallmark gene sets were tested to define biological programs associated with this macrophage state.

### Cell–cell communication analysis

Ligand-receptor analysis was used to infer communication among epithelial, stromal, lymphoid, and myeloid compartments. Cell-type labels from the integrated single-cell object served as input. Communication probabilities were calculated from curated ligand-receptor pairs, and cell populations with insufficient representation were excluded when necessary to avoid unstable estimates. Global interaction networks were visualized to compare communication patterns across cell types.

Mac_APOE cells were analyzed as both sender and receiver populations. Differential interaction strength between primary and metastatic lesions was calculated, and selected ligand-receptor pairs involving Mac_APOE and epithelial cells were visualized with heatmaps and dot plots.

### Untargeted metabolomics sequencing

Tissue samples were thawed on ice and extracted with precooled 80% methanol. For each sample, 50 mg of tissue was homogenized in 0.5 mL of 80% methanol, incubated at -20 °C for 30 min, and centrifuged at 20, 000 × g for 15 min. The supernatant was vacuum-dried, reconstituted in 100 μL of 80% methanol, and stored at -80 °C before liquid chromatography-mass spectrometry (LC-MS) analysis. Pooled quality control (QC) samples were prepared by combining 10 μL of supernatant from each of the 120 extracts.

Metabolites were detected with a Q Exactive LC-MS system (Thermo Scientific) in positive and negative ionization modes. Precursor ions were scanned over m/z 70-1050, and fragment ion scans were acquired according to the instrument method. A QC sample was injected after every 10 samples. Raw MS data were processed with XCMS and R-based workflows to generate a three-dimensional data matrix. Metabolites were annotated against Kyoto Encyclopedia of Genes and Genomes (KEGG), Human Metabolome Database (HMDB), and an in-house library using a mass tolerance of <10 ppm. Differential metabolites were used for KEGG/Gene Set Enrichment Analysis (GSEA) enrichment and pathway-network analysis.

### Cell lines and *Bacillus* supernatant preparation

SW480 (CVCL_0546) and SW620 (CVCL_0547) human CRC cell lines were purchased from the Cell Resource Center of the Chinese Academy of Sciences. Cells were maintained in RPMI 1640 medium supplemented with 10% fetal bovine serum (FBS) at 37 °C in a humidified incubator with 5% CO2. The cells used in this study tested negative for mycoplasma, and neither cell line appears on the International Cell Line Authentication Committee list of commonly misidentified cell lines.

The *Bacillus* strain was obtained from the Beina Biological Fungi and Bacteria Preparation Center (BNCC337007). Bacteria were inoculated into LB medium and cultured at 37 °C with shaking at 200 r/min for 24 h. Cultures were centrifuged at 8000 r/min for 10 min, and the supernatant was collected and filtered through a 0.22-μm membrane before use.

### Animal experiments

Male BALB/c nude mice aged 6 weeks were purchased from Jiangsu Jicui Yaoke Biotechnology Co., Ltd. and housed in the Animal Experiment Center of the Tenth People’s Hospital Affiliated with Tongji University. A CRLM model was established by intrasplenic injection of 1 × 10^6^ SW480Luc cells in 100 μL into each mouse.

Mice were randomly assigned to five groups with five mice per group: Ctrl, Bac-Live, Bac-SN, Bac-Live + Abx IV, and Bac-Live + Abx DW. The Ctrl group received PBS and SW480Luc cells. The Bac-Live group received viable *Bacillus* and SW480Luc cells. The Bac-SN group received *Bacillus* supernatant and SW480Luc cells. The Bac-Live + Abx IV group received viable *Bacillus*, SW480Luc cells, and intravenous (IV) Abx. The Bac-Live + Abx DW group received viable *Bacillus*, SW480Luc cells, and Abx administered in drinking water (DW).

For DW antibiotic treatment, vancomycin (0.5 mg/mL), imipenem/cilastatin (0.5 mg/mL), neomycin (1 mg/mL), and amphotericin (0.5 mg/mL) were dissolved in sterile water and supplied ad libitum until the endpoint. Bottles and antibiotic solutions were replaced every other day because imipenem/cilastatin has a short half-life. For IV treatment intended to target intratumoral microbiota while preserving gut microbiota, vancomycin (10 mg/mL), imipenem/cilastatin (4 mg/mL), and neomycin (1.5 mg/mL) were injected through the tail vein. Each mouse received 300 μL once every 48 h until 48 h before endpoint analysis, following a previously described approach (14).

On day 14 after intervention, tumor bioluminescence was measured with an *in vivo* imaging system. On day 28, mice were euthanized by slow-fill carbon dioxide inhalation at a chamber displacement rate of 30% per min, followed by cervical dislocation to confirm death. Liver tissues were dissected and collected for evaluation of hepatic metastatic tumor formation.

### Bacterial preparation

The *Bacillus* strain was isolated and identified by the Beina Biological Fungi and Bacteria Preparation Center. It was inoculated into LB medium, incubated with shaking at 37 °C and 200 r/min for 24 hours, and then centrifuged at 8000 r/min for 10 min to collect the supernatant. The supernatant was sterilized by filtration through a 0.22 μm membrane, yielding the *Bacillus* supernatant.

### Wound healing assay

SW480 and SW620 cells were seeded in six-well plates and grown to approximately 90% confluence. A vertical scratch was generated with a sterile 200 μL pipette tip. Detached cells were removed by three PBS washes, and serum-free medium containing *Bacillus* supernatant prepared at the indicated multiplicity of infection (MOI)-equivalent conditions was added. Images were collected at 0 and 48 h with an inverted microscope. Wound closure was quantified with ImageJ.

### Transwell migration assay

Transwell inserts were placed in 24 well plates. The upper chamber contained serum-free medium with *Bacillus* supernatant and 5 × 10^4^ SW480 or SW620 cells, and the lower chamber contained 600 μL of medium supplemented with 10% FBS. After 48 h at 37 °C with 5% CO2, nonmigrated cells on the upper membrane surface were removed. Migrated cells were fixed with 4% paraformaldehyde for 30 min, stained with 0.1% crystal violet for 20 min, rinsed with PBS, and air-dried. Five fields of view were imaged for each insert, and ImageJ was used for quantification.

### CCK-8 proliferation assay

SW480 and SW620 cells were seeded into 96-well plates at 1 × 10^3^ cells per well and cultured for 24 h. *Bacillus* supernatant at different MOI-equivalent conditions was then added. Cell proliferation was measured at 0, 24, 48, 72, 96, and 120 h. At each time point, 10 μL of CCK-8 reagent was added to each well, and the plates were incubated at 37 °C for 2 h. Absorbance at 450 nm was recorded with a microplate reader and used to estimate proliferative activity.

### Statistical analysis

Continuous variables were analyzed with nonparametric tests and are presented as medians with interquartile ranges. Categorical variables were compared with the chi-square test, and Fisher’s exact test was used when any expected cell count was less than 5. Spearman rank correlation analysis was used to assess associations between microbial abundance, metabolites, and clinical variables. The significance threshold was α = 0.05, with P < 0.05 considered statistically significant. Heatmaps and statistical plots were generated in R. Figure labels indicate *P < 0.05, **P < 0.01, and ***P < 0.001.

## Results

### Patient cohort and sample classification

After 105 patients were screened, 20 patients with non-metastatic CRC and 20 patients with CRLM were included. In the CRLM cohort, CRLM-C referred to primary colorectal lesions and CRLM-L referred to paired liver metastatic lesions. Tumor tissues were labeled T, and adjacent nontumor tissues were labeled P. The final six sample groups were CRC-T, CRC-P, CRLM-C-T, CRLM-C-P, CRLM-L-T, and CRLM-L-P. **Supplementary Table 1** summarizes the clinical characteristics of the enrolled patients.

### 5R 16S rDNA sequencing defines microbial differences between primary and metastatic lesions

**Figure 1:**
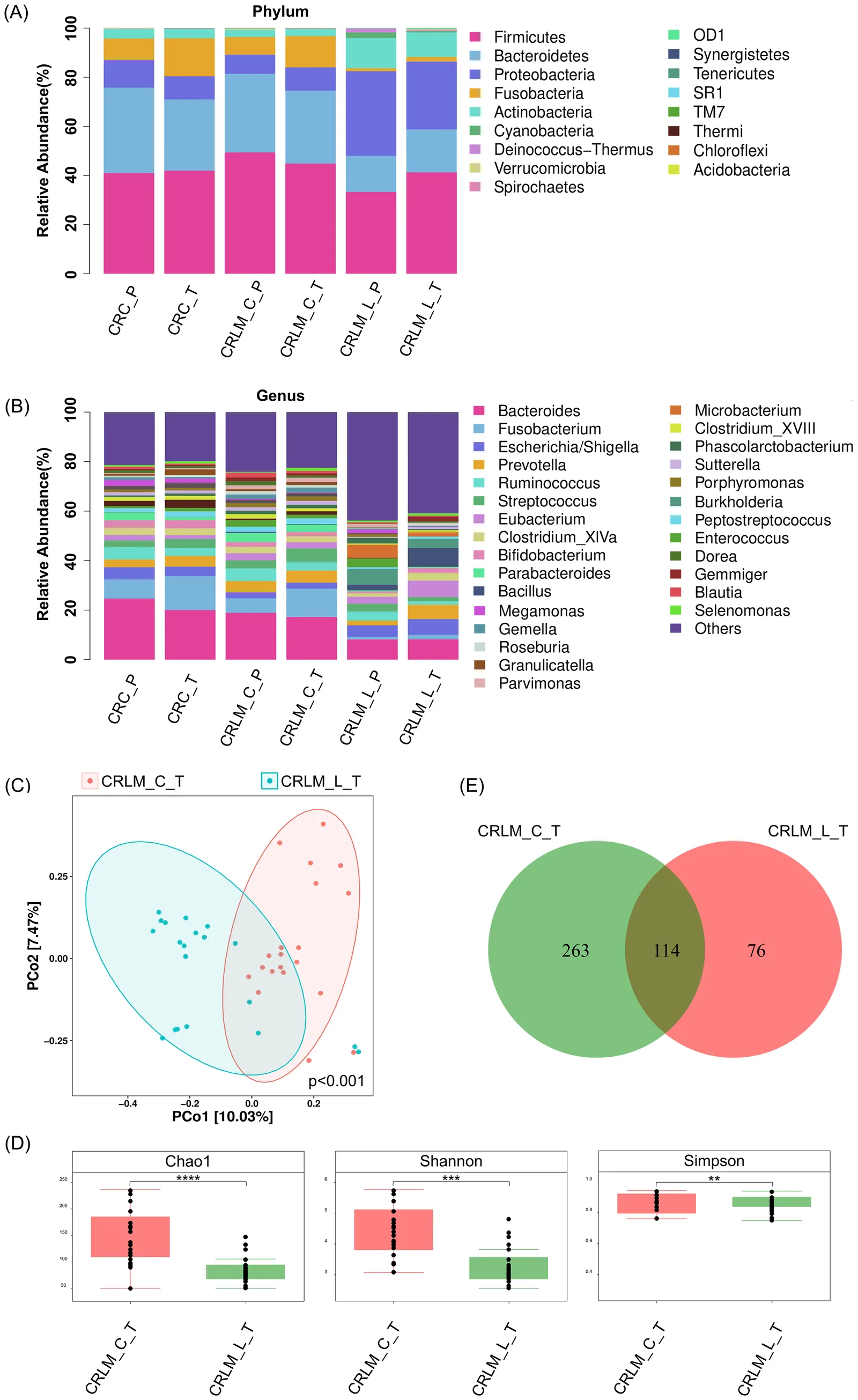
Intratumoral microbial composition and diversity in CRC and CRLM samples. **(A)** Relative abundance of major bacterial phyla across sample groups. **(B)** Genus-level microbial composition across groups. **(C)** PCoA comparing CRLM-C-T and CRLM-L-T microbial profiles. **(D)** Alpha diversity indices in CRLM-C-T and CRLM-L-T samples. **(E)** Genus-level Venn diagram comparing CRLM-C-T and CRLM-L-T groups. CRC-T, tumor lesions from patients with primary colorectal cancer; CRLM-C-T, primary tumor lesions from patients with colorectal liver metastasis; CRLM-L-T, liver metastatic lesions from patients with colorectal liver metastasis. **P < 0.01; ***P < 0.001; ****P < 0.0001.

5R 16S rDNA sequencing was used to compare intratumoral microbial communities across the six sample groups. At the phylum level, *Firmicutes*, *Bacteroidetes*, *Proteobacteria*, *Fusobacteria*, and *Actinobacteria* were the main taxa detected across groups (**Figure 1A**), broadly consistent with previous descriptions of tumor-associated microbial communities (15–17). At the genus level, *Bacteroides*, *Fusobacterium*, *Escherichia*, and *Prevotella* were the dominant genera (**Figure 1B**).

Principal coordinate analysis (PCoA) showed separation between CRLM-C-T and CRLM-L-T samples (P < 0.001; **Figure 1C**). Other group comparisons did not show significant separation (**Supplementary Figure 1A**). Chao1, Shannon, and Simpson indices were higher in CRLM-C-T samples than in CRLM-L-T samples (**Figure 1D**), while alpha diversity did not differ significantly among the other groups (**Supplementary Figure 1B**). The genus-level Venn diagram showed 114 shared genera, 263 genera detected only in primary CRLM tumors, and 76 genera detected only in liver metastases (**Figure 1E**). Primary and paired liver metastatic lesions therefore showed site-associated microbial differences, whereas tumor and adjacent tissues within the same anatomical context showed less pronounced separation.

### *Bacillus* is enriched in liver metastatic lesions and linked to predicted lipid metabolism

**Figure 2:**
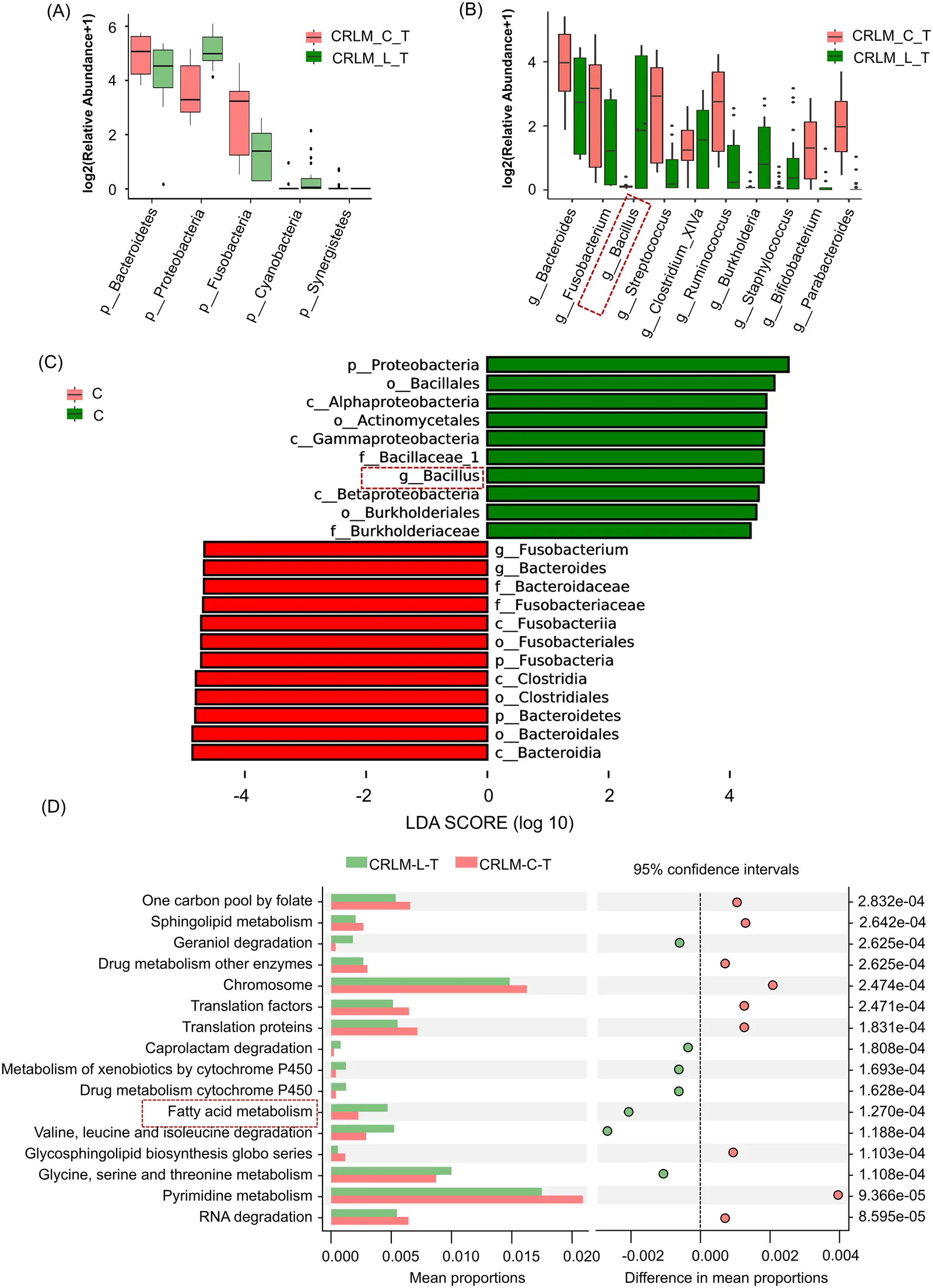
Differential microbial taxa and predicted microbial functions in paired CRLM lesions. **(A)** Phylum-level taxa differing between CRLM-C-T and CRLM-L-T groups. **(B)** Top genus-level differences between the two groups; **Supplementary Figure 2A** shows the full list of 73 differentially abundant genera. **(C)** LEfSe analysis of intratumoral microbiota in primary and metastatic lesions. **(D)** PICRUSt2-based KEGG level 3 pathway prediction comparing microbial functional profiles between the two groups.

We next examined taxa that distinguished CRLM primary tumors from paired liver metastases. Phylum-level analysis showed site-associated microbial differences (**Figure 2A**). *Proteobacteria* and *Cyanobacteria* were more abundant in CRLM-L-T samples, whereas *Bacteroidetes*, *Fusobacteria*, and *Synergistetes* were more abundant in CRLM-C-T samples. At the genus level, 73 bacterial genera differed between the two groups. *Bacillus* showed the highest enrichment in CRLM-L-T samples (P = 0.0059; **Figure 2B**; **Supplementary Figure 2A**). LEfSe analysis and cladogram visualization supported the taxonomic enrichment of *Bacillus* in liver metastatic lesions (**Figure 2C**; **Supplementary Figure 2B**).

PICRUSt2-based KEGG level 3 prediction indicated functional divergence between primary and metastatic microbial communities. CRLM-C-T samples were enriched for pyruvate metabolism, sphingolipid metabolism, and one-carbon pool by folate. CRLM-L-T samples were enriched for fatty acid metabolism, geraniol degradation, and valine, leucine, and isoleucine degradation (**Figure 2D**). Because *Bacillus* has been linked to bacterial and host lipid metabolic regulation (18, 19), the enrichment of *Bacillus* in CRLM-L-T samples pointed to a possible connection between metastasis-associated bacteria and fatty-acid-related pathways.

### Liver metastatic lesions show accumulation of lipid and bile-acid metabolites

**Figure 3:**
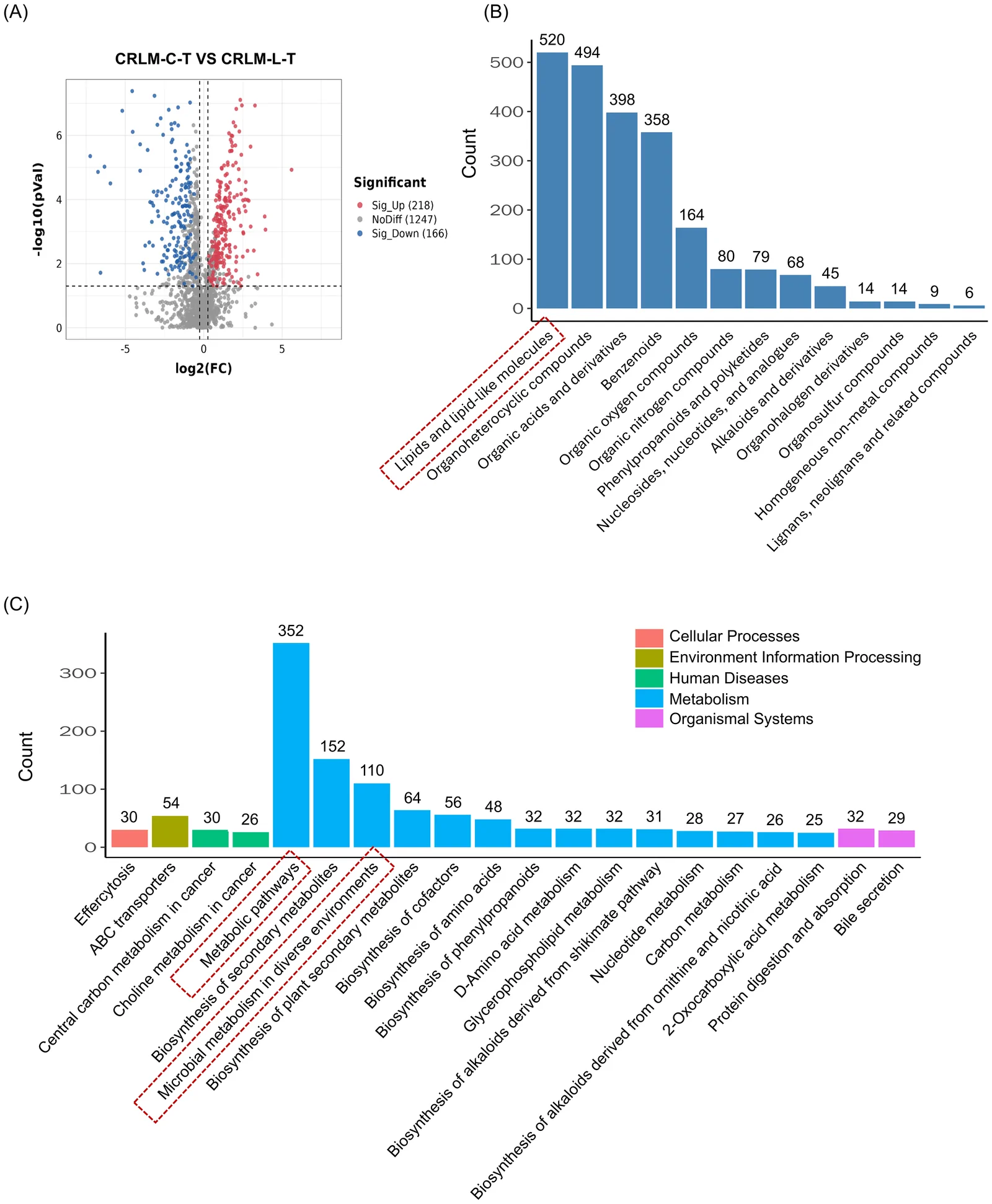
Metabolomic differences between CRLM primary tumors and liver metastases. **(A)** Volcano plot of differential metabolites between CRLM-C-T and CRLM-L-T groups. **(B)** HMDB superclass annotation of differential metabolites. **(C)** KEGG level 3 pathway annotation of differential metabolites.

Untargeted metabolomics was performed to determine whether microbial differences were accompanied by metabolic changes. The comparison between CRLM-C-T and CRLM-L-T samples identified 384 differential metabolites, including 218 increased and 166 decreased metabolites in CRLM-L-T samples (**Figure 3A**). Lipid-related molecules accounted for a large portion of the differential metabolites, including carnitine-related metabolites such as decanoyl-L-carnitine and 2-octenoyl-L-carnitine, and bile-acid-related metabolites such as glycoursodeoxycholic acid (**Supplementary Figure 3A**).

HMDB superclass annotation showed that lipids and lipid-like molecules formed the largest detected category, with 520 annotated features (**Figure 3B**). KEGG annotation placed metabolism as the main enriched functional category, with bile secretion and glycerophospholipid metabolism among the annotated pathways (**Figure 3C**). The enrichment of microbial metabolism in diverse environments also suggested a relationship between intratumoral microbial structure and the tissue metabolic state. Metabolite-pathway networks connected differential carnitine- and bile-acid-related metabolites with fatty acid transport and lipid degradation pathways (**Supplementary Figure 3B**). These metabolomic data were consistent with the microbial functional prediction pointing to fatty acid metabolism in liver metastatic lesions.

### *Bacillus* abundance correlates with tumor markers and lipid-related metabolites

**Figure 4:**
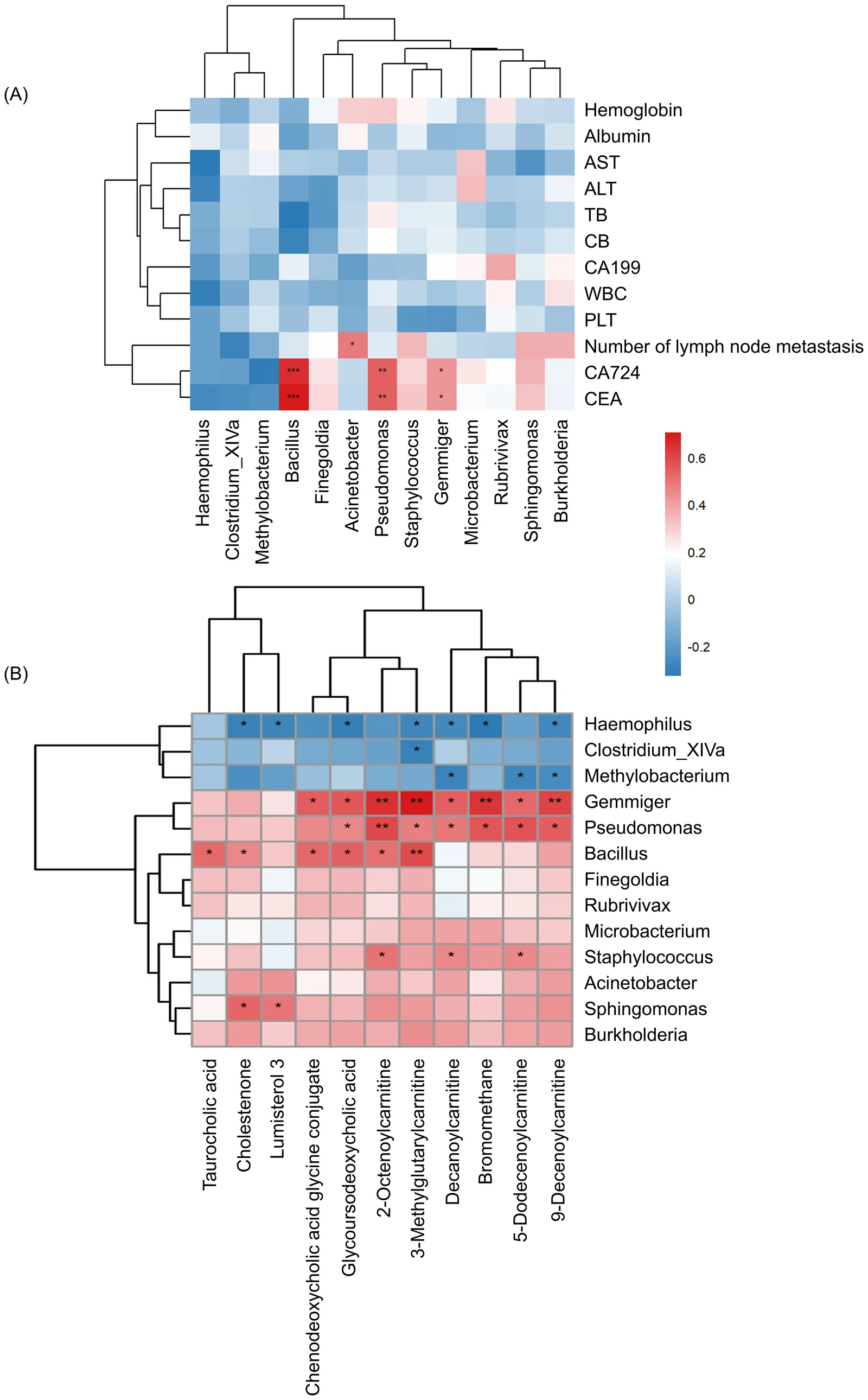
Associations among metastasis-enriched bacteria, lipid metabolites, and clinical indicators. **(A)** Correlation heatmap between differential bacterial genera and clinical indicators, including HB, albumin, AST, ALT, TB, CB, CA19-9, CA724, CEA, WBC, PLT, and lymph node metastasis number. **(B)** Correlation heatmap between differential bacterial genera and metabolites enriched in CRLM-L-T samples. Colors indicate correlation coefficients. *P < 0.05; **P < 0.01; ***P < 0.001.

We then evaluated the clinical and metabolic associations of metastasis-enriched microbes. *Bacillus*, *Gemmiger*, and *Pseudomonas* showed positive correlations with carcinoembryonic antigen (CEA) and CA19–9 levels (P < 0.05). *Bacillus* showed the strongest relationship with tumor markers among these taxa (r = 0.588, P < 0.001; **Figure 4A**).

*Bacillus* also correlated positively with several metabolites enriched in CRLM-L-T samples, including 3-methylglutarylcarnitine, 2-octenoylcarnitine, glycoursodeoxycholic acid, chenodeoxycholic acid glycine conjugate, cholestenone, and taurocholic acid (P < 0.05; **Figure 4B**). The correlation pattern placed *Bacillus* at the intersection of metastasis-associated microbial enrichment, tumor-marker elevation, and lipid-metabolite accumulation.

### *Bacillus* supernatant enhances CRC cell migration and proliferation *in vitro*

**Figure 5:**
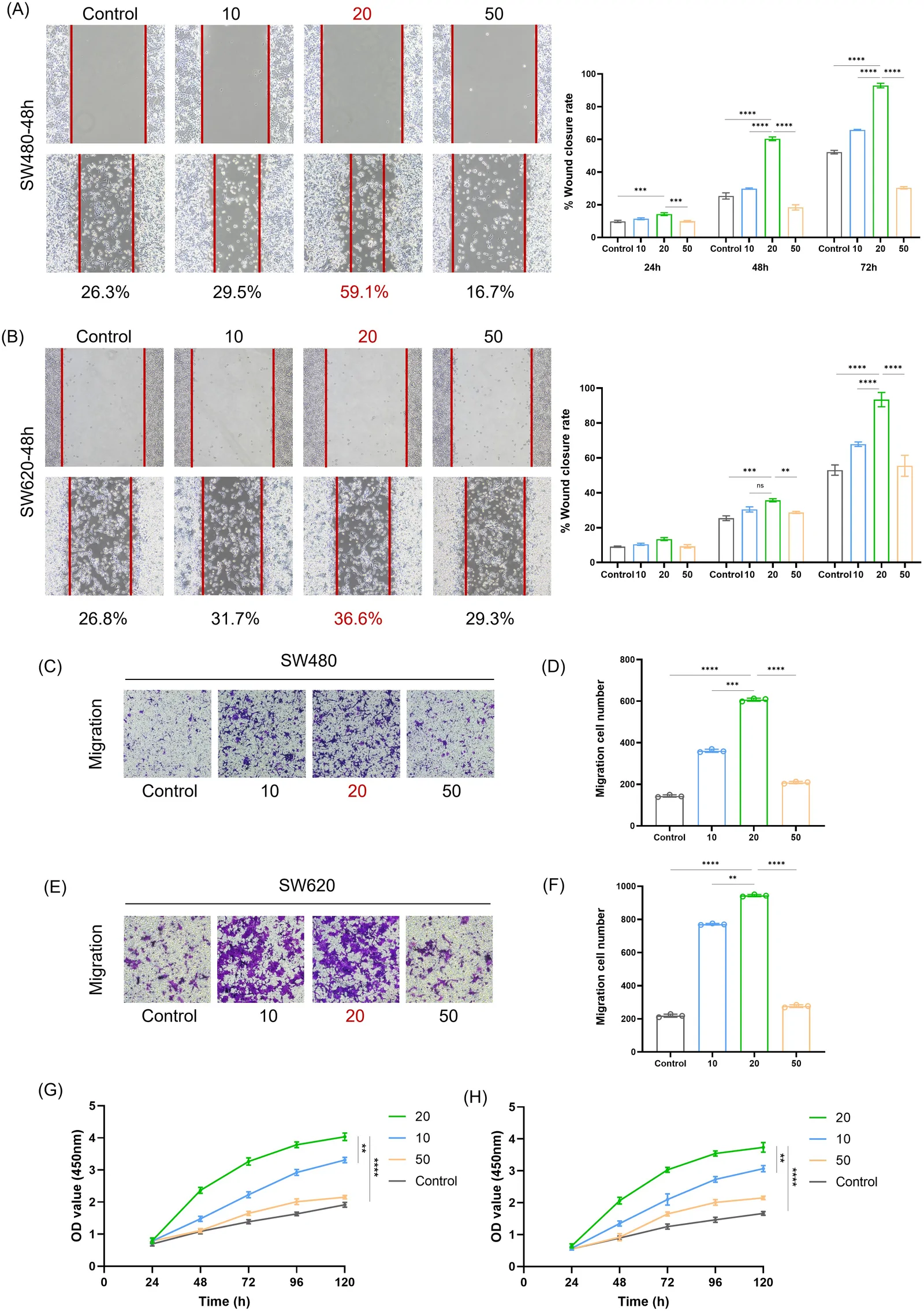
*Bacillus* supernatant enhances CRC cell migration and proliferation *in vitro*. **(A, B)** Wound healing assays in SW480 **(A)** and SW620 **(B)** cells treated with *Bacillus* supernatant; values in parentheses indicate wound closure rates. **(C, D)** Transwell migration assays in SW480 cells. **(E, F)** Transwell migration assays in SW620 cells. **(G, H)** CCK-8 proliferation assays in SW480 and SW620 cells treated with *Bacillus* supernatant. **P < 0.01; ***P < 0.001; ****P < 0.0001; ns, not significant.

Direct exposure to viable *Bacillus* caused contamination-related stress and death in CRC cell cultures, so *Bacillus* supernatant was used to test effects on tumor cell behavior. SW480 and SW620 cells were treated with *Bacillus* supernatant prepared under MOI-equivalent conditions of 0, 10, 20, and 50. In wound healing assays, the supernatant increased cell migration in a concentration-dependent pattern up to MOI 20. The MOI 20 condition produced the highest wound closure rates in both SW480 cells (59.1% vs. 26.3%) and SW620 cells (36.6% vs. 26.8%) compared with control conditions (**Figures 5A, B**). At MOI 50, wound closure declined to 16.7% in SW480 cells and 29.3% in SW620 cells.

Transwell assays showed the same direction of change, with increased migration of SW480 and SW620 cells after treatment with *Bacillus* supernatant and the clearest effect at MOI 20 (**Figures 5C–F**). CCK-8 assays showed higher proliferative activity over five days after *Bacillus* supernatant exposure (**Figures 5G, H**). MOI 20 consistently produced higher OD450 values than the other conditions (P < 0.05). MOI 10 and MOI 50 also increased cell viability, but their effects were lower than that observed with MOI 20.

### *Bacillus* enhances metastatic tumor growth *in vivo*

An intrasplenic SW480Luc CRLM model was used to test whether *Bacillus* affected metastatic tumor growth *in vivo*. Five intervention groups were established: Ctrl, Bac-Live, Bac-SN, Bac-Live + Abx IV, and Bac-Live + Abx DW. The Ctrl group received PBS and SW480Luc cells. Bac-Live and Bac-SN groups received viable *Bacillus* or *Bacillus* supernatant with SW480Luc cells. The two antibiotic groups received viable *Bacillus* and SW480Luc cells together with IV Abx or antibiotic-containing DW.

**Figure 6:**
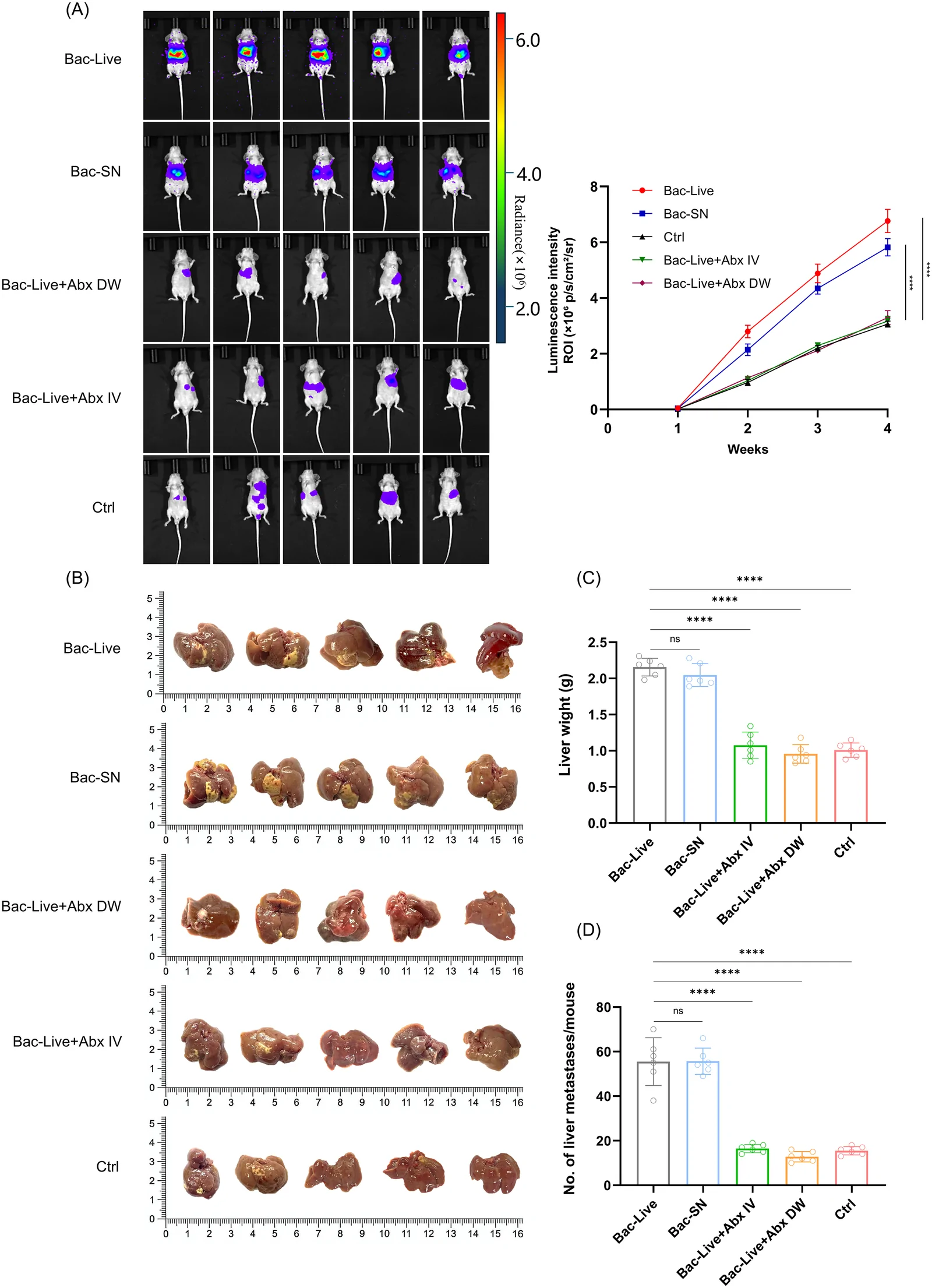
*Bacillus* enhances metastatic tumor growth *in vivo*. **(A)** *In vivo* bioluminescence imaging of metastatic tumor signals across groups. **(B)** Representative anatomical images of mouse livers. **(C)** Liver weight across groups. **(D)** Number of liver metastatic nodules per mouse. A CRLM mouse model was generated by intrasplenic injection of SW480Luc cells. Mice were assigned to Ctrl, Bac-Live, Bac-SN, Bac-Live + Abx IV, and Bac-Live + Abx DW groups. ****P < 0.0001; ns, not significant.

Bioluminescence imaging showed higher tumor signals in Bac-Live and Bac-SN mice than in the other groups (**Figure 6A**). Antibiotic treatment through either IV injection or DW reduced the bioluminescent signal toward the control range. Liver metastatic nodule counts and liver burden measurements were consistent with the imaging results (**Figures 6C, D**). These animal data indicate that *Bacillus*-associated signals increased metastatic tumor growth in this model, and antibiotic intervention attenuated this effect.

### Single-cell analysis reveals TIME remodeling in CRLM

**Figure 7:**
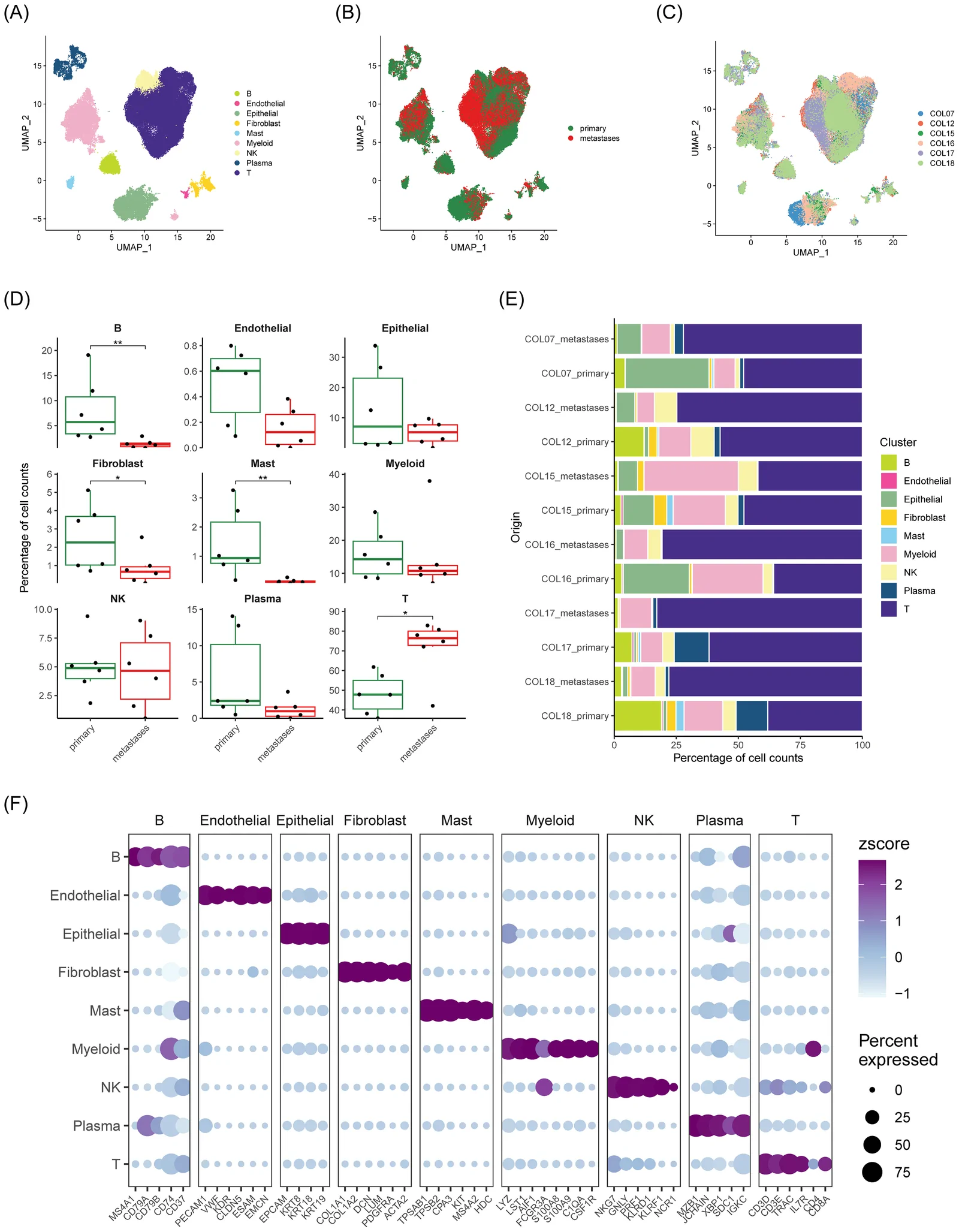
Single-cell profiling reveals TIME remodeling in CRLM. **(A)** UMAP plot of major cellular compartments, including B cells, endothelial cells, epithelial cells, fibroblasts, mast cells, myeloid cells, NK cells, plasma cells, and T cells. **(B)** UMAP visualization colored by anatomical origin. **(C)** UMAP visualization colored by individual patient sample. **(D)** Relative proportions of major cell types in primary and metastatic lesions. **(E)** Stacked barplot showing cell population percentages across individual primary and metastatic samples. **(F)** Dot plot of canonical lineage marker expression used for cell-type annotation. *P < 0.05; **P < 0.01.

To connect microbial and metabolic alterations with host-cell remodeling, we analyzed scRNA-seq profiles from primary colorectal tumors and liver metastatic lesions. After QC, integration, and clustering, major cell populations were annotated as T cells, myeloid cells, epithelial cells, fibroblasts, endothelial cells, B cells, plasma cells, NK cells, and mast cells (**Figure 7A**). Projection by anatomical origin showed that primary and metastatic lesions shared the same major lineages, but metastatic samples occupied a different cellular composition within the shared transcriptional space (**Figure 7B**). Sample level UMAP visualization also showed patient to patient heterogeneity while preserving the overall cellular architecture (**Figure 7C**).

Cell-composition analysis showed remodeling of the TIME in liver metastases. Compared with primary tumors, metastatic lesions contained a higher proportion of T cells and lower proportions of B cells, fibroblasts, and mast cells (**Figure 7D**). Epithelial, endothelial, myeloid, NK, and plasma-cell compartments varied across patients but did not show a uniform depletion pattern. Stacked barplot analysis confirmed that liver metastases were not simply immune-cold lesions; instead, they showed immune and stromal reorganization with prominent T-cell infiltration and persistent myeloid representation (**Figure 7E**). Canonical marker expression supported the cell-type annotations (**Figure 7F**).

### APOE^+^ macrophages exhibit lipid-associated features and inferred communication patterns

**Figure 8:**
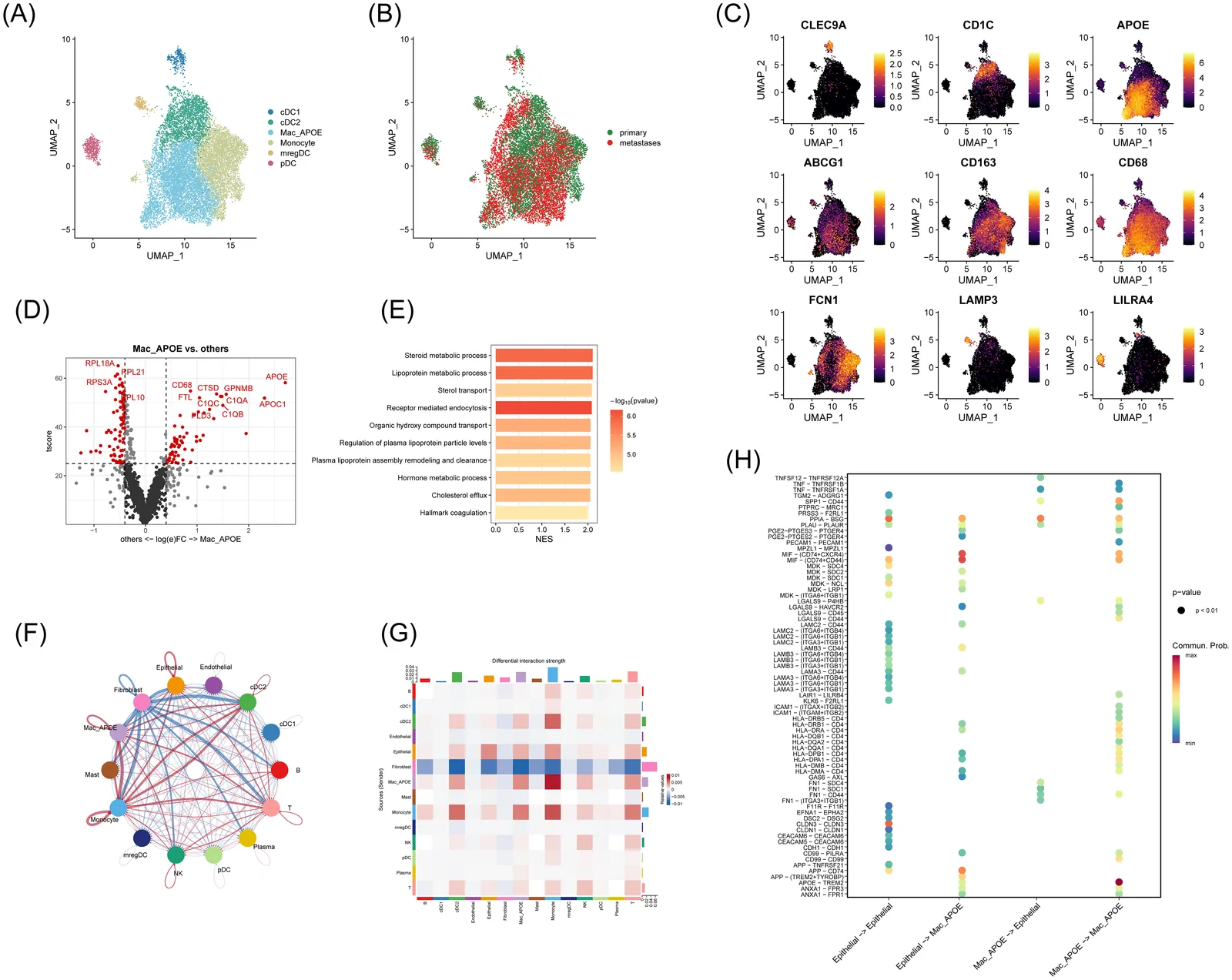
APOE^+^ macrophages represent a lipid-associated myeloid state with inferred communication patterns. **(A)** UMAP plot of myeloid subclusters, including cDC1, cDC2, Mac_APOE, monocytes, mature regulatory DCs, and pDCs. **(B)** Myeloid UMAP colored by anatomical origin. **(C)** Feature plots of canonical myeloid and lipid-handling markers, including CLEC9A, CD1C, APOE, ABCG1, CD163, CD68, FCN1, LAMP3, and LILRA4. **(D)** Differential expression analysis comparing Mac_APOE cells with other myeloid subsets. **(E)** Biological pathways enriched among genes upregulated in Mac_APOE cells. **(F)** Global ligand-receptor communication network across major cell compartments, highlighting Mac_APOE connectivity. **(G)** Heatmap of differential interaction strength across cell populations. **(H)** Dot plot of ligand-receptor interaction probabilities between Mac_APOE cells and epithelial cells.

We next reanalyzed the myeloid compartment. Subclustering identified cDC1, cDC2, monocytes, LAMP3^+^ mature regulatory DCs, pDCs, and an APOE^+^ macrophage population designated Mac_APOE (**Figure 8A**). Projection by tissue origin showed that these myeloid states appeared in both primary and metastatic lesions, suggesting remodeling of shared myeloid programs rather than replacement by a lesion-specific myeloid lineage (**Figure 8B**).

Marker expression supported the myeloid annotations. CLEC9A marked cDC1, CD1C marked cDC2, FCN1 marked monocytes, LAMP3 marked mature regulatory DCs, and LILRA4 marked pDCs. Mac_APOE cells expressed APOE, ABCG1, CD163, and CD68 (**Figure 8C**). Differential expression analysis showed enrichment of APOE, APOC1, C1QA, C1QB, C1QC, CD68, CTSD, GPNMB, and FTL in Mac_APOE cells, consistent with a complement-rich and metabolically active macrophage state (**Figure 8D**).

Functional enrichment analysis of Mac_APOE-upregulated genes identified lipid- and sterol-related programs, including steroid metabolic process, lipoprotein metabolic process, sterol transport, receptor-mediated endocytosis, regulation of plasma lipoprotein particle levels, plasma lipoprotein assembly, remodeling and clearance, and cholesterol efflux (**Figure 8E**). The Mac_APOE phenotype therefore provided a host-cell correlate for lipid metabolic regulation in liver metastatic lesions.

Cell-cell communication analysis predicted broad interactions among epithelial, stromal, lymphoid, and myeloid compartments. Mac_APOE cells occupied a highly connected position in the inferred communication network (**Figure 8F**). Differential interaction analysis suggested increased communication involving myeloid populations, particularly monocytes and Mac_APOE cells, whereas fibroblast-associated interactions were reduced (**Figure 8G**). Focused ligand-receptor analysis predicted potential interactions involving APOE-TREM2, APP-TNFRSF21, ANXA1-FPR1, MIF-CD74/CXCR4, SPP1-CD44, LGALS9-HAVCR2, HLA class II-CD4, CD99-CD99, laminin-integrin, and ICAM1-integrin interactions involving Mac_APOE and epithelial or immune compartments (**Figure 8H**). These predicted interaction patterns suggest that APOE^+^ macrophages may represent highly connected nodes in the inferred communication network.

## Discussion

Intratumoral microbiota has been increasingly recognized as an active component of the tumor microenvironment, with roles in tumor initiation, progression, immune regulation, and metastasis (17, 20, 21). Our comparison of primary and paired metastatic CRLM lesions extends this concept to the liver metastatic niche. The separation between CRLM-C-T and CRLM-L-T microbial profiles suggests that metastatic lesions do not simply preserve the primary tumor microbial state. Instead, the hepatic environment appears to select for a narrower microbial community. The present study examined this concept in CRLM by integrating 5R 16S rDNA sequencing, untargeted metabolomics, single-cell transcriptomics, and functional validation. The data showed that primary colorectal lesions and paired liver metastatic lesions differed in microbial diversity and taxonomic composition. *Bacillus* was enriched in liver metastatic tumors, correlated with serum CEA and CA19–9 levels, and was associated with lipid-related metabolites that accumulated in metastatic lesions. *Bacillus* supernatant promoted CRC cell migration and proliferation *in vitro*, and viable *Bacillus* or *Bacillus* supernatant increased metastatic tumor growth *in vivo*. Antibiotic intervention reduced this effect. Single-cell analysis further identified APOE^+^ macrophages as lipid-associated myeloid cells with broad communication with epithelial, immune, and stromal compartments. These observations reveal associations among *Bacillus* enrichment, lipid-related metabolic alterations, and APOE^+^ macrophage-associated communication signatures in CRLM.

The microbial differences between primary and metastatic lesions suggest that the metastatic site may imposes selective pressure on tumor-associated bacteria. Previous studies have shown that intratumoral microbiota varies across tumor types, anatomical sites, and spatial niches within tumors (22). Pan-cancer analysis of metastatic tumors has also demonstrated that metastatic lesions may harbor microbiota patterns distinct from those of primary tumors (21). In liver-related tumor settings, intratumoral microbial composition has been linked to local immune and metabolic features, supporting the view that the hepatic microenvironment can shape microbial colonization (23). Our observation that liver metastatic lesions had lower microbial alpha diversity than paired primary tumors is consistent with this site-filtering concept. The intestine contains a dense and diverse microbial reservoir, whereas the liver exposes incoming microbes to bile acids, resident macrophages, and sinusoidal immune surveillance (24–27). These hepatic barriers may eliminate many intestinal bacteria while permitting selected organisms with higher stress tolerance to persist.

*Bacillus* enrichment in liver metastatic lesions may reflect this selective niche pressure. *Bacillus* species are facultative anaerobes widely present in the environment, fermented foods, and the gastrointestinal tract, and several *Bacillus* strains have been studied as probiotics (28–33). *Bacillus* endospores can tolerate harsh environmental conditions, which may help explain why this genus can survive in restrictive microenvironments (34). At the same time, *Bacillus* is not uniformly beneficial. Certain *Bacillus* species, including *B. cereus*, have been associated with opportunistic infection and bacteremia, showing that members of this genus can exert harmful effects under specific host conditions (35, 36). The current study did not resolve *Bacillus* at species or strain level. Therefore, the data should be interpreted at the genus level, and future work should determine whether specific *Bacillus* species or bacterial products are responsible for the observed prometastatic effects.

The functional data support a role for *Bacillus*-derived signals in CRC cell behavior. *Bacillus* supernatant increased SW480 and SW620 cell migration and proliferation, with the strongest activity at MOI 20. The reduced effect at higher MOI suggests that *Bacillus*-derived factors may have concentration-dependent and potentially non-linear biological effects. We speculated that moderate concentrations may provide an optimal level of prometastatic factors, whereas higher concentrations may contain cytostatic or cytotoxic components that counteract the migration- and proliferation-promoting effects. Liu et al. investigated the effects of lipopeptides produced by *Bacillus subtilis* HSO121 on Bcap-37 breast cancer cells and found that, once the lipopeptide concentration reached a specific physicochemical threshold, its predominant mode of action could shift from cellular signaling modulation to pronounced membrane damage and direct cell lysis (37). This finding provides biological plausibility for the attenuated effect observed at the higher concentration in our study.

The *in vivo* data provided further support, as both viable *Bacillus* and *Bacillus* supernatant increased metastatic tumor burden in the splenic injection model. Antibiotic treatment reduced the tumor-promoting effect, which indicates that bacterial depletion can alter the metastatic phenotype. Similar observations have been reported for other CRC-associated bacteria. *Fusobacterium nucleatum* promotes CRC progression in specific genetic contexts, *Escherichia coli* has been linked to metastatic regulation, and *Streptococcus gallolyticus*-derived factors can influence macrophage polarization and inflammatory signaling (38–40). These studies place *Bacillus* within a broader group of tumor-associated bacteria that can modify CRC progression, although the molecular mechanisms appear to differ among bacterial taxa. It was worth noting that since the experimental strain was not isolated from CRLM tissues, we were unable to establish strain-level correspondence with the intratumoral *Bacillus* detected in patient samples. However, previous studies have similarly used authenticated commercial strain to evaluate the potential biological effects of tumor-associated bacteria under controlled conditions (41, 42). The authenticated commercial strain provided a standardized, traceable, and reproducible model for functional evaluation.

The metabolomic results provide a second layer of evidence connecting *Bacillus* enrichment with the liver metastatic niche. Liver metastatic lesions accumulated lipid and lipid-like metabolites, including carnitine-related and bile-acid-related metabolites. PICRUSt2 analysis also suggested enrichment of fatty acid metabolism-related microbial functions in liver metastases. These findings are consistent with recent studies showing that tumor-associated bacteria can alter host lipid metabolism. *Gordonia polyisoprenivorans* was reported to promote cervical cancer progression through lysoPC-mediated lipid metabolic disruption, and ATF6-dependent lipid remodeling in CRC was shown to select for tumor-associated *Desulfovibrio (*43, 44). *Bacillus* species have also been linked to lipid metabolic regulation in experimental models. *Bacillus licheniformis* FA6 affected lipid metabolism by promoting acetyl-CoA synthesis and inhibiting β-oxidation, while *Bacillus amyloliquefaciens* TL regulated lipid-anabolic programs through the IGF signaling pathway (45, 46). These studies support the plausibility of a *Bacillus*–lipid metabolism connection, but the present data remain correlative at the metabolite level. Conversely, the lipid-rich metabolic environment of liver metastatic lesions may favor the enrichment or persistence of *Bacillus*, indicating that the observed metabolic alterations may not be solely driven by *Bacillus*-associated effects. Therefore, the directionality of the relationship between *Bacillus* enrichment and lipid-related metabolic alterations cannot be determined from the current data. Bacterial metabolite tracing, *Bacillus*-conditioned metabolomics, and targeted lipid rescue experiments will be required to establish direct causality.

The single-cell analysis extended the microbial and metabolic findings into the host immune compartment. CRLM liver metastases did not show a simple immune-cold pattern. Instead, metastatic lesions displayed broad TIME remodeling, with altered proportions of T cells, B cells, fibroblasts, mast cells, and myeloid populations. This pattern fits the current view that CRLM progression involves plasticity of tumor cells and reciprocal communication with the hepatic niche rather than passive tumor-cell dissemination alone (2–4). The myeloid compartment was particularly relevant in our analysis. APOE^+^ macrophages expressed APOE, APOC1, complement genes, CD68, CTSD, GPNMB, and FTL, and their enriched pathways involved cholesterol efflux, sterol transport, lipoprotein metabolism, and receptor-mediated endocytosis. These features identify Mac_APOE cells as lipid-associated macrophages located within a metastasis-adapted immune microenvironment.

APOE^+^ macrophages may provide a host-cell link between lipid metabolic regulation and immune regulation in CRLM. Macrophage polarization has been implicated in CRC progression, and immunosuppressive macrophage states can support tumor growth and immune escape (47). The hepatic microenvironment also contains specialized macrophage and stromal populations that influence tissue injury, inflammation, and malignant transformation (48, 49). In the present study, Mac_APOE cells occupied a potential position in the inferred communication network and interacted with epithelial, stromal, lymphoid, and other myeloid compartments. Ligand–receptor pairs such as APOE–TREM2, ANXA1–FPR1, APP–TNFRSF21, MIF–CD74/CXCR4, SPP1–CD44, LGALS9–HAVCR2, and laminin–integrin axes suggest potential programs related to lipid sensing, macrophage adaptation, immune modulation, and epithelial interaction, while additional spatial and functional validation will be required to confirm the biological relevance of these interactions. These inferred interactions do not prove direct cellular signaling, but they identify candidate pathways through which lipid-associated macrophages may shape the metastatic niche.

The clinical correlation analysis adds translational relevance to the microbial findings. *Bacillus* abundance was positively correlated with serum CEA and CA19-9, two markers commonly used in gastrointestinal tumor monitoring. A previous study in intrahepatic cholangiocarcinoma reported associations between intratumoral bacterial taxa, including *B. anthracis* and *Pseudomonas azotoformans*, and CA19–9 levels (50). In CRC, *Fusobacterium nucleatum* abundance increases during tumor progression, contributes to chemotherapy resistance through TLR4/NF-κB/BIRC3 signaling, and may influence prognosis in defined clinical subgroups (51–53). *Peptostreptococcus anaerobius* can also promote resistance to anti-PD-1 therapy by shaping a myeloid-derived suppressor cell (MDSC)-dependent immunosuppressive environment (54). Broader microbiome-based clustering studies have linked colorectal tissue microbiota patterns with patient prognosis (55). These studies suggest that tumor-associated bacteria may have value as biomarkers or therapeutic modifiers. In the present study, *Bacillus* should be regarded as a candidate metastasis-associated genus rather than an established clinical biomarker.

The antibiotic experiments indicate that microbial targeting can modify *Bacillus*-associated metastatic growth, but the therapeutic implications require caution. Broad-spectrum antibiotic treatment may affect intratumoral bacteria, gut microbiota, systemic immunity, hepatic microbial exposure, and host metabolism simultaneously. The reduction of tumor burden after antibiotic intervention therefore cannot be attributed exclusively to *Bacillus* depletion. The difference between IV and DW antibiotic strategies provide useful information, but both approaches remain relatively non-specific. Future studies should use species-level *Bacillus* isolation, defined bacterial recolonization, bacterial product fractionation, and narrow-spectrum or engineered bacterial targeting strategies to determine whether *Bacillus* can be selectively and safely manipulated in CRLM.

Several limitations should be considered. First, the clinical cohort was limited in size, and multicenter validation is needed to confirm *Bacillus* enrichment, microbial diversity changes, and metabolite associations in independent CRLM populations. Second, although DNA extraction blanks and PCR negative controls were included, an independent tissue-processing blank control was not available; therefore, potential contamination introduced during tissue collection, storage, or pre-extraction processing cannot be completely excluded. Third, the metabolomic analysis showed lipid accumulation in liver metastatic lesions, but the study did not directly trace whether these metabolites originated from bacteria, tumor cells, hepatocytes, or immune cells. Fourth, the single-cell analysis was based on public data and computational inference of ligand–receptor interactions. Spatial validation, multiplex immunostaining, and co-culture experiments are needed to confirm the localization and function of APOE^+^ macrophages. Fifth, the animal model demonstrated *Bacillus*-associated enhancement of metastatic growth, but additional models are required to distinguish effects on tumor seeding, hepatic colonization, and metastatic outgrowth.

In summary, the present study characterizes CRLM-associated microbial, metabolic, and immune pattern characterized by *Bacillus* enrichment, lipid metabolite accumulation, and APOE^+^ macrophage-centered communication. The data reveal concurrent microbial, metabolic, and immune alterations in which metastasis-enriched *Bacillus* is linked to lipid metabolic regulation and myeloid adaptation within the liver metastatic niche. Further mechanistic studies are needed to identify the *Bacillus* species, bacterial products, lipid mediators, and macrophage pathways. This work provides a basis for evaluating intratumoral microbiota and lipid-associated macrophages as potential biomarkers or therapeutic vulnerabilities in CRLM.

## Conclusion

This study identifies microbial, metabolic, and immune differences between colorectal primary tumors and paired liver metastatic lesions. *Bacillus* was enriched in liver metastases, correlated with tumor markers and lipid-related metabolites, and promoted CRC cell migration, proliferation, and metastatic tumor growth in experimental models. Metabolomic analysis showed lipid and bile-acid metabolite accumulation in liver metastatic tissues. Single-cell analysis identified APOE^+^ macrophages as lipid-associated myeloid cells with broad inferred communication activity in the CRLM microenvironment. These results support potential associations among microbial, metabolic, and immune features in CRLM progression and suggest that *Bacillus*-associated pathways and APOE^+^ macrophage communication may represent therapeutic vulnerabilities that require further validation.

## Data Availability

The datasets presented in this study can be found in online repositories. The names of the repository/repositories and accession number(s) can be found below: CRA045349 (https://ngdc.cncb.ac.cn/gsa/browse/CRA045349).
