# Supplementary material for: Intratumoral Bacillus regulating colorectal cancer liver metastasis: links to lipid metabolism and APOE+ macrophages
Source: Front Immunol. 2026 Aug 28;17:1938606. doi: 10.3389/fimmu.2026.1938606 (PMC13561942; doi:10.3389/fimmu.2026.1938606)
Supplement: Supplementary file 1 [file SupplementaryFile1.docx]

**FIGURE S1. Diversity of the intratumoral** **microbiota between tumor and adjacent nontumor tissues.** (A) PCoA plots showing Bray-Curtis based beta diversity across four groups comparisons. (B) Alpha diversity of four groups comparisons.

**FIGURE S2. Differential intratumoral microbiota composition between primary and liver metastatic lesions.** (A) Bar plot showing log₂-transformed relative abundance of the top bacterial genera between the two groups. (B) LEfSe cladogram illustrating taxa with significantly differential abundance between the two groups.

**FIGURE S3. Differential metabolites and functional pathway enrichment between primary and liver metastatic lesions.** (A) Box plots showing relative abundance of significantly differentially expressed metabolites between the two groups. (B) Metabolic pathway enrichment network based on differentially expressed metabolites.
