# Supplementary material for: Intratumoral Bacillus regulating colorectal cancer liver metastasis: links to lipid metabolism and APOE+ macrophages
Source: Front Immunol. 2026 Aug 28;17:1938606. doi: 10.3389/fimmu.2026.1938606 (PMC13561942; doi:10.3389/fimmu.2026.1938606)
Supplement: Supplementary file 2 [file SupplementaryFile2.docx]

Supplementary Table 1. Demographic and clinical characteristics of the study patients.

| **Variabl****e** | **non-metastatic CRC (n=20)**  **Number (%) / median (IQR)** | **CRLM (n=20)**  **Number (%) / median (IQR)** | **P** |
| --- | --- | --- | --- |
| Age | 56 (46-63) | 56 (48-66) | 0.415 |
| Gender |  |  |  |
| Male | 12 (60) | 13 (65) | 0.744 |
| Female | 8 (40) | 7 (35) |  |
| Body mass index | 23.7 (19.8-28.1) | 23.4 (19.9-27.8) | 0.872 |
| Diabetes |  |  |  |
| Yes | 5 (25) | 4 (20) | 0.705 |
| No | 15 (75) | 16 (80) |  |
| Hypertension |  |  |  |
| Yes | 8 (40) | 7 (35) | 0.744 |
| No | 12 (60) | 13 (65) |  |
| Chronic Liver Disease |  |  |  |
| Yes | 2 (10) | 3 (15) | 0.674 |
| No | 18 (90) | 17 (85) |  |
| CEA (ng/mL) | 12.2 (1.7-20.9) | 81.7 (19.3-205.4) | <0.001 |
| CA199 (U/mL) | 13.5 (1.4-21.3) | 53.5 (14.0-368.2) | 0.001 |
| CA724 (U/mL) | 7.1 (1.6-17.7) | 9.4 (2.1-18.5) | 0.486 |
| AFP (ng/mL) | 3.1 (1.6-5.7) | 3.1 (1.3-4.9) | 0.904 |
| Site of Primary Tumor |  |  |  |
| Ascending colon | 4 (20) | 3 (15) | 0.972 |
| Descending colon | 5 (25) | 6 (30) |  |
| Sigmoid colon | 5 (25) | 5 (25) |  |
| Rectum | 6 (30) | 6 (30) |  |
| Tumor Grade |  |  |  |
| Moderately differentiated | 19 (95) | 13 (65) | 0.028 |
| Poorly differentiated | 1 (5) | 7 (35) |  |
| Stage AJCC |  |  |  |
| I-II | 11 (55) | 0 (0) | <0.001 |
| III | 9 (45) | 0 (0) |  |
| IV | 0 (0) | 20 (100) |  |
| Characteristics of liver metastases |  |  |  |
| Solitary metastasis | NA | 16 (80) | - |
| Multiple metastases | NA | 4 (20) | - |
| Largest liver metastasis diameter (cm) | NA | 2.5 (1.7-3.8) | - |
| Unilobar distribution | NA | 17 (85) | - |
| Bilobar distribution | NA | 3 (15) | - |
